# Modeling of clinical phenotypes in systemic lupus erythematosus based on the platelet transcriptome and FCGR2a genotype

**DOI:** 10.1186/s12967-023-04059-w

**Published:** 2023-04-07

**Authors:** MacIntosh G. Cornwell, Hanane El Bannoudi, Elliot Luttrell-Williams, Alexis Engel, Tessa J. Barrett, Khrystyna Myndzar, Peter Izmirly, H. Michael Belmont, Robert Clancy, Kelly V. Ruggles, Jill P. Buyon, Jeffrey S. Berger

**Affiliations:** 1 Division of Precision Medicine, Department of Medicine, New York University Grossman School of Medicine, New York, NY USA; 2 Institute for Systems Genetics, New York University Grossman School of Medicine, New York, NY USA; 3 Division of Cardiology, Department of Medicine, New York University Grossman School of Medicine, New York, NY USA; 4 Division of Rheumatology, Department of Medicine, New York University Grossman School of Medicine, NYU Grossman School of Medicine, Medical Science Building 593, 530 First Avenue, New York, NY 10016 USA; 5 Center for the Prevention of Cardiovascular Disease, New York University Grossman School of Medicine, 530 First Avenue, Skirball 9R, New York, NY 10016 USA

**Keywords:** Platelet, Lupus, RNA-seq, Transcriptomics, FCGR2a, Lupus Nephritis, Systemic lupus erythematosus

## Abstract

**Background:**

The clinical heterogeneity of SLE with its complex pathogenesis remains challenging as we strive to provide optimal management. The contribution of platelets to endovascular homeostasis, inflammation and immune regulation highlights their potential importance in SLE. Prior work from our group showed that the Fcγ receptor type IIa (FcγRIIa)–R/H131 biallelic polymorphism is associated with increased platelet activity and cardiovascular risk in SLE. The study was initiated to investigate the platelet transcriptome in patients with SLE and evaluate its association across FcγRIIa genotypes and distinct clinical features.

**Methods:**

Fifty-one patients fulfilling established criteria for SLE (mean age = 41.1 ± 12.3, 100% female, 45% Hispanic, 24% black, 22% Asian, 51% white, mean SLEDAI = 4.4 ± 4.2 at baseline) were enrolled and compared with 18 demographically matched control samples. The FCGR2a receptor was genotyped for each sample, and RNA-seq was performed on isolated, leukocyte-depleted platelets. Transcriptomic data were used to create a modular landscape to explore the differences between SLE patients and controls and various clinical parameters in the context of FCGR2a genotypes.

**Results:**

There were 2290 differentially expressed genes enriched for pathways involved in interferon signaling, immune activation, and coagulation when comparing SLE samples vs controls. When analyzing patients with proteinuria, modules associated with oxidative phosphorylation and platelet activity were unexpectedly decreased. Furthermore, genes that were increased in SLE and in patients with proteinuria were enriched for immune effector processes, while genes increased in SLE but *decreased* in proteinuria were enriched for coagulation and cell adhesion. A low-binding FCG2Ra allele (R131) was associated with decreases in FCR activation, which further correlated with increases in platelet and immune activation pathways. Finally, we were able to create a transcriptomic signature of clinically active disease that performed significantly well in discerning SLE patients with active clinical disease form those with inactive clinical disease.

**Conclusions:**

In aggregate, these data demonstrate the platelet transcriptome provides insight into lupus pathogenesis and disease activity, and shows potential use as means of assessing this complex disease using a liquid biopsy.

**Supplementary Information:**

The online version contains supplementary material available at 10.1186/s12967-023-04059-w.

## Background

Systemic Lupus Erythematosus (SLE) is often referred to as an archetypical example of an autoimmune disease. The pathology is still not fully understood due to the complexity of the underlying mechanism of disease, which is further complicated by its heterogenous clinical phenotypes. Over two decades ago it was noted that the majority of deaths in patients with longstanding SLE were attributed to cardiovascular disease (CVD) [1]. Neither traditional cardiovascular risk factors nor SLE-associated risk factors such as corticosteroid treatment, antiphospholipid (aPL) antibodies and the ongoing inflammatory process entirely account for the increased cardiovascular risk [1–4]. Current evaluation of SLE using the hybrid SELENA SLEDAI (Safety of Estrogens in Lupus National Assessment–Systemic Lupus Erythematosus Disease Activity Index) evaluates patients using a points per domain criteria [5–7]. While this current assessment measure provides a valuable tool for tracking disease changes, molecular based subtyping of the pathophysiology of SLE is still not used clinically. The use of novel biospecimens in combination with sequencing analysis has potential for understanding the interplay between cardiovascular risk and SLE.

Platelets, long established as more than anucleate cell fragments whose sole purpose is clotting, play key roles in inflammatory and immune mediated diseases [8]. While several studies have demonstrated that platelet activity is increased in SLE [8–10], the precise mechanism of how SLE activated platelets contribute to the pathogenesis of SLE tissue inflammation and injury is unclear. Platelets contain transcripts and the necessary molecular machinery to conduct translation and are intercellular regulators of inflammation and immune activation. Activated platelets can stimulate endothelial cells, neutrophils, and monocytes to produce inflammatory cytokines and chemokines [11–13]. Platelet transcriptomics have been used to identify individuals with metabolic, inflammatory, and cardiovascular diseases [9, 12, 14–16]. Thus, through their genotype, phenotype, and complex network of effects, platelets may be a significant mediator of SLE disease activity and life-threatening comorbidities.

In addition to the well described heightened platelet activity in SLE, it is necessary to determine how platelets interact with their environment and confer their effect onto surrounding cells [9]. FcγRIIA, encoded by The *FCGR2A* gene, is a platelet receptor that interacts with immunoglobulin and immune complexes and mediates interactions with the platelet’s microenvironment and surrounding immune cells [17]. The receptor displays a functional allelic dimorphism generating two codominantly expressed allotypes with either a histidine (H) or an arginine (R) at amino acid position 131 in the second Ig-like extracellular domain [18]. These variants confer functional significance since they differ in their ability to bind IgG-containing immune complexes: the 131H allotype efficiently binds human IgG1 and IgG2, whereas the 131R allotype binds human IgG1 but poorly binds IgG2 and as such the variants are referred to as high-binding and low-binding, respectively [19]. With regard to antibody-mediated disease such as SLE, the consequences of this differential binding capacity have demonstrated clinical importance, as the expression and response to FcγRIIA signaling pathways are associated with lupus nephritis [20], and the low binding 131R allele is associated with increased platelet activity and subclinical CVD [21]. Despite these studies, the specific mechanism by which FcγRIIA confers CVD risk is still unknown, and elucidating its role in platelets and vascular health should contribute to our understanding of its role in disease.

Accordingly, this study was designed to better evaluate the role of platelets in SLE. We first characterized the platelet transcriptome across all samples using an unsupervised approach followed by the identification of significant molecular differences seen between SLE and controls. Using a modular transcriptomic framework, differences were evaluated across SLE disease activity, and in participants with active proteinuria, identifying both genes and pathways involved in disease pathogenesis. Further, the effects of FcγRIIA variants on the platelet transcriptome were investigated both in healthy controls and participants with SLE. Lastly, we establish a groundwork for the exploration of platelet transcriptomic profiles and their potential use in predicting changes in lupus activity over time.

## Methods

### Patient population

Patients were recruited from the NYU Lupus Cohort. The NYU Lupus Cohort is a prospective convenience registry open to enrollment of any patient with SLE seen at NYU Langone Health and Bellevue Hospital Center since 2014. All SLE patients in the NYU Lupus Cohort are age 18 or older and fulfill at least one of the following criteria for SLE: (1) the American College of Rheumatology (ACR) revised classification criteria [22, 23]; (2) the Systemic Lupus International Collaborating Clinics (SLICC) classification criteria [24]; (3) the European League Against Rheumatism (EULAR)/ACR classification criteria [25]. All patients and controls signed an informed consent (available in English, Spanish, and Mandarin) approved by the NYU and Bellevue Institutional Review Boards. Exclusion from this study included the use of any dose of aspirin and/or an anticoagulant, a platelet count < 100 X 10^9^ g/l, and a hemoglobin level < 9 g/dl. Despite these criteria, three patients were enrolled with a platelet count < 100 X 10^9^ g/l, but quality control of data showed no specific reason for exclusion in this analysis.

At each patient visit, overall disease activity was measured by the hybrid SELENA SLEDAI [5–7] (Buyon, Thanou) which incorporates the definition of the proteinuria domain used in the SLEDAI 2 K (Gladman), namely protein > 500 mg/d (UPCR > 0.5) is always scored regardless of whether it is persistent. To evaluate clinical phenotypes, patients were assigned more granularly to one of 8 groups based on dominance of laboratory and or specific organ involvement (Table 1). For each patient a disease activity “dominant” category was also assigned. The categories were defined based on the scoring of each domain with a hierarchy as follows: inactive (0 points SLEDAI), serologically active no clinical disease (2–4 points, low C, positive anti-dsDNA, either or both), hematologic (1–6 points, low White Blood Cell or platelets plus or minus serologic activity), serositis (2–10 points, pleuritis, pericarditis, either or both plus or minus serologic activity plus or minus hematologic activity), musculoskeletal (4–14 points, > 2 synovitis in 2 or more joints, plus or minus any other of the above domains), active nephritis (4–26 points, uPCR > 0.5 with our without urine WBC,RBC, or casts plus or minus any of the above), and finally, CNS disease of SLEDAI > 20. Medication use and dosages were also collected at each visit: glucocorticoids, hydroxychloroquine (HCQ), immunosuppressants including azathioprine, mycophenolate mofetil (MMF), methotrexate, belimumab, tacrolimus.

**Table 1:** Description of cohort

Category	Feature	Control (N = 18)	SLE (N = 51)	p. value
Age	mean (± sd)	42.06 (15.65)	41.14 (12.35)	0.823
Gender	Female, N (%)	18 (100)	51 (100)	1
Ethnicity	Hispanic/Latino, N (%)	4 (22.2)	23 (45.1)	0.071
Race	Asian, N (%)	1 (5.6)	12 (23.5)	0.132
	Black or African American, N (%)	6 (33.3)	12 (23.5)	
	White, N (%)	10 (55.6)	27 (52.9)	
	More than one race, N (%)	1 (5.6)	0 (0)	
Genotype	Homo ancestral, N (%)	4 (22.2)	12 (23.5)	0.993
	Hetero variant, N (%)	8 (44.4)	22 (43.1)	
	Homo variant, N (%)	6 (33.3)	17 (33.3)	
SLE Only				
		N available	N (%)	Dose (± sd)
Medication	Prednisone, N (%), mg	51	12 (23.5)	6.88 (3.04)
	Hydroxychloroquine, N (%), mg	51	41 (80.4)	351.22 (84.03)
	Azathioprine, N (%), mg	51	5 (9.8)	85 (33.54)
	Mycophenolate mofetil N (%), mg	51	13 (25.5)	2269.23 (753.20)
	Methotrexate, N (%), mg	51	2 (3.9)	13.75 (8.84)
	Benlysta, N (%), IV	51	4 (7.8)	NA
SELENA SLEDAI Domains	Seizure	51	0 (0)	
	Psychosis	51	0 (0)	
	Organic brain syndrome	51	0 (0)	
	Visual disturbance	51	0 (0)	
	Cranial nerve disorder	51	0 (0)	
	Lupus headache	51	0 (0)	
	Cerebrovascular accident	51	0 (0)	
	Vasculitis	51	0 (0)	
	Arthritis	51	2 (4.1)	
	Myositis	51	0 (0)	
	Hematuria	50	4 (8.0)	
	Proteinuria	51	13 (25.5)	
	Pyuria	51	3 (5.9)	
	Rash	51	8 (15.7)	
	Alopecia	51	4 (7.8)	
	Mucosal ulcers	51	0 (0)	
	Pleurisy	51	0 (0)	
	Pericarditis	51	0 (0)	
	Low complement	51	25 (49)	
	Increased DNA binding	49	23 (46.9)	
	Fever	51	0 (0)	
	Thrombocytopenia	50	3 (6)	
	Leukopenia	51	5 (9.8)	
SLEDAI Total	mean (± sd)	51	4.24 (4.12)	
Predominant Disease Category	Active lupus nephritis, N (%)	47	13 (27.7)	
	Active musculoskeletal disease, N (%)	47	2 (4.3)	
	Predominately active cutaneous disease, N (%)	47	4 (8.5)	
	Predominately hematologic activity, N (%)	47	7 (14.9)	
	Clinically inactive disease and either ds-DNA or low complement N (%)	47	13 (27.7)	
	Clinically and serologically inactive disease, N (%)	47	8 (17)	

Descriptive characteristics of the SLE and control cohort. In addition, for the SLE cohort, ACR criteria, SLEDAI domains, medication usage, predominant disease category and progression status are also included. Percentages are based on available assessment data

### Platelet RNA isolation and library preparation

Platelets were isolated from blood collected in tubes containing 3.2% sodium citrate, after a 2 cc discard. After a 15 min rest, the blood was centrifuged at 200 g for 10 min to obtain platelet rich plasma (PRP). Following addition of Acid citrate dextrose (ACD) to the PRP in a 1:10 ratio and a 10 min rest, the platelets were subjected to centrifugation at 1000 g for 10 min. The pellet was resuspended in EasySep^™^ Buffer (STEMCELL Technologies) and incubated with microbeads to deplete leukocytes and red blood cells using EasySep^™^ Human CD45 Depletion Kit II and EasySep^™^ RBC Depletion Reagent respectively (STEMCELL Technologies). Efficiency of CD45 + cell and red blood cell depletion was confirmed by flow cytometry at the end of the isolation protocol. (Additional file 1: Fig S1). Isolated platelets were lyzed in 500 μL QIAzol^™^ (Qiagen) and stored at − 80 °C until use.

RNA was extracted using Direct-zol MicroPrep Kits (ZYMO RESEARCH) according to manufacturer’s instructions. RNA quality and quantity were determined using an Agilent 2100 Bioanalyzer (Agilent Technologies). Sequencing libraries were generated with Clontech SMART-Seq HT kit (Takara Bio) for low input mRNA per manufacturer’s instructions. The obtained libraries were quantified, normalized, pooled, and sequenced using the S1 100 flow cell on NovaSeq 6000 instrument (Illumina).

### FCγRIIA genotyping

FCγRIIA genotyping was determined in purified DNA samples using Applied Biosystems^™^ TaqMan^™^ SNP Genotyping Assay (SNP ID: rs1801274, Assay ID: C___9077561_20) and TaqMan^™^ Genotyping Master Mix (Thermo Fisher). The obtained results were analyzed using allelic exclusion technique on Applied Biosystems^™^ Genotyping Analysis software.

### Data preprocessing

FASTQ files from RNA-sequencing were processed using the Seq-N-Slide pipeline [26]. Briefly, reads quality was assessed using FASTQC [27], fastqscreen [28], and picard [29]. Samples were then aligned to the hg38 genome using STAR [30], and reads were quantified using featurecounts [31].

### Bioinformatic and statistical analyses

All analyses were performed in R. Differential expression analyses were all performed using DESeq2 [32]. All figures including heatmaps, scatterplots, boxplots, and upset plots were created using a combination of ggplot2 [33] and ComplexHeatmap [34]. The modular gene framework was created using Weighted Gene Co-expression Network Analysis (WGCNA) [35]. WGCNA eigengene values are the first principal component for the expression of the module, and are conceptualized as the directional relative co-expression of the entire module. Annotation of our modular gene network was performed using hypergeometric Gene Set Enrichment Analysis (GSEA) tests using Gene Ontology (GO) terms from the Molecular Signatures Database (MsigDB). Gene Set Enrichment Analysis (GSEA) [36] for our differentially expressed genes was performed using clusterprofiler, [37] and enriched for pathways provided by MSigDB [38] via the msigdbr [39] package. The Disease Activity Progression score was derived using singscore [40]. Briefly, singscore calculates stable expression based score for single samples using a ranked expression table and an “upSet” for upregulated genes of interest and a “downSet” for downregulated genes of interest. The single score is the “TotalScore” which is the aggregate measure of expression of both the up and down gene sets.

For machine learning for prediction of SLE we used five different ML models: (1) Generalized Linear Model (GLM), (2) xgBoost, (3) SVM with a radial kernel, (4) Random Forest, and (5) Lasso. For each of these tests, we measured performance success using the Area Under the Curve (AUC).

All pairwise statistical tests were performed using a Welch’s *t*-test unless otherwise noted. All correlational tests were performed using the nonparametric Spearman correlation unless otherwise noted. Multiple hypothesis correction was performed where applicable using the Benjamini–Hochberg method [41].

## Results

### Initiation and implementation of a platelet transcriptomic modular framework

**Fig. 1:**
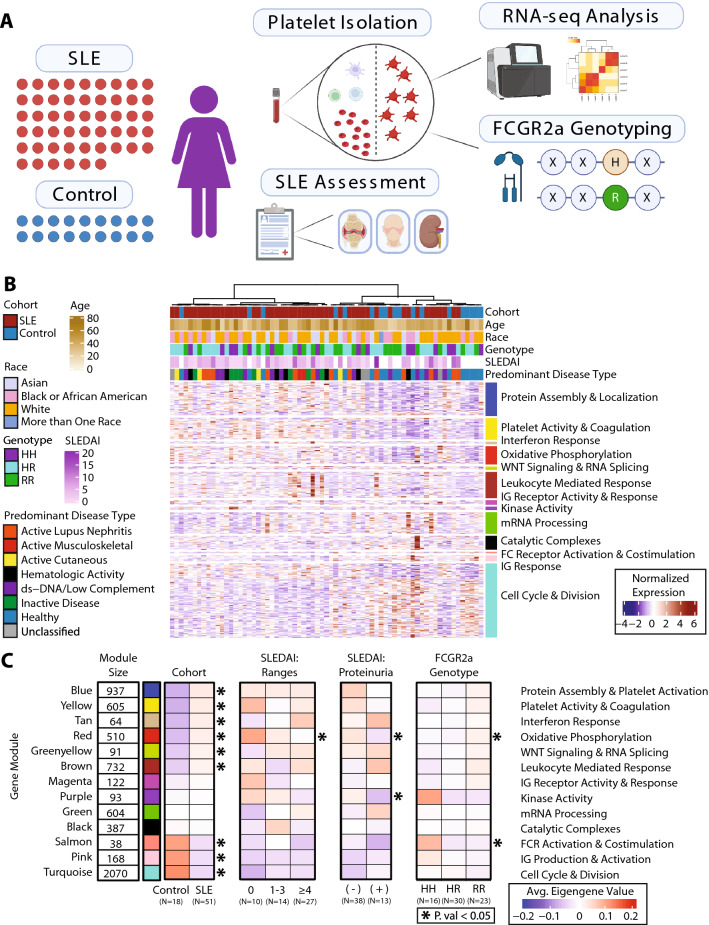
Initiation and Implementation of a Platelet Transcriptomic Modular Framework. **A** Schematic of the cohort and workflow. 51 SLE samples and 18 control samples were collected, and patients were assessed on SLE criteria and other baseline characteristics. Platelets were extracted from blood samples, and RNA-sequencing and FCGR2a genotyping was performed. **B** Heatmap of our RNA-seq cohort with samples along the x-axis and genes along the y-axis. Samples are annotated along the horizontal axis for cohort, age, race, FcγRIIA genotype, and SLEDAI and predominant disease category for SLE patients. Clustering of samples was performed using the top 20% of varied genes, the genes in the heatmap are those that composed the gene modules as determined by WGCNA. **C** Reduction of our RNA-seq cohort down to average eigengene values per subgroup. In order: The Cohort columns show the average eigengene value for patients in our Control subgroup and SLE subgroup. The SLEDAI Ranges columns show the average eigengene value for our SLE patients within each range of SLEDAI values (0, 1–3, ≥ 4). The SLEDAI Proteinuria columns show the average eigengene value for our SLE patients who have proteinuria (UPCR > 0.5) and those who do not. The FcγRIIA genotype columns show the average eigengene value for patients with each respective FcγRIIA genotype (HH, HR, RR). Cohort comparisons with a statistical test’s p value < 0.05 are denoted with an asterisk. Pairwise comparisons (Cohort, Proteinuria) were performed using a *t*-test and threeway comparisons (SLEDAI Ranges, FcγRIIA genotype) were performed using an ANOVA test

RNA-sequencing was performed on platelets isolated from 51 subjects with SLE (100% Female, Age: 41.4 ± 13.2 years, 26.1% Black, 18.8% Asian, 53.6% White) and 18 controls (100% Female, Age: 42.06 ± 15.65 years, 33.3% Black, 5.6% Asian, 55.6% White). (Table 1, Fig. 1A). Reads were processed, quantified, and then further filtered for significantly expressed genes, resulting in 9769 genes for downstream analysis. (Additional file 1: Fig S2A). Using the top 20% of varied genes, unsupervised hierarchical clustering was applied to sort the samples. The resulting heatmap showed separation between participants with SLE and controls. (Fig. 1B).

Next, using Weighted Gene Co-expression Network Analysis (WGCNA), we aimed to reduce our genes down to a set of co-regulated modules to explore for specific signals that would be consistent across our SLE population, and to find signals with higher variance within our heterogenous population. The resulting modules are 13 blocks of genes ranging in size from 38 to 2070 genes and are each denoted by a specific color. The eigengene expression values from these modules are representative of the relative co-expression of the entire block of genes. We enriched for genes across 14,998 GO (Gene Ontology) terms [42] and selected a representative descriptive term for each module based on the GSEA (Gene Set Enrichment Analysis) results (Additional file 2: Table S1, Additional file 1: Fig S2B). Reducing our module’s expression to a single eigengene value, we summarized the expression of each module per sample. Clustering across all samples using this framework yielded robust separation between participants with SLE and controls. (Additional file 1: Fig S2C).

Using this modular framework, the population was divided into various subgroups to evaluate global transcriptomic differences in the platelets isolated from subjects segregated by different clinical characteristics. This applied framework is summarized in Fig. 1C, where we divided our population along several axes to address the following comparisons: (1) SLE vs controls, (2) SLE across ranges of disease activity as measured by SLEDAI (3) SLE with and without proteinuria (defined using a standard measure of a Urine Protein: Creatinine Ratio (UPCR) of > 0.5), and (4) high vs low binding FcγRIIA genotype [19].

### The platelet transcriptome is significantly altered in SLE

**Fig. 2:**
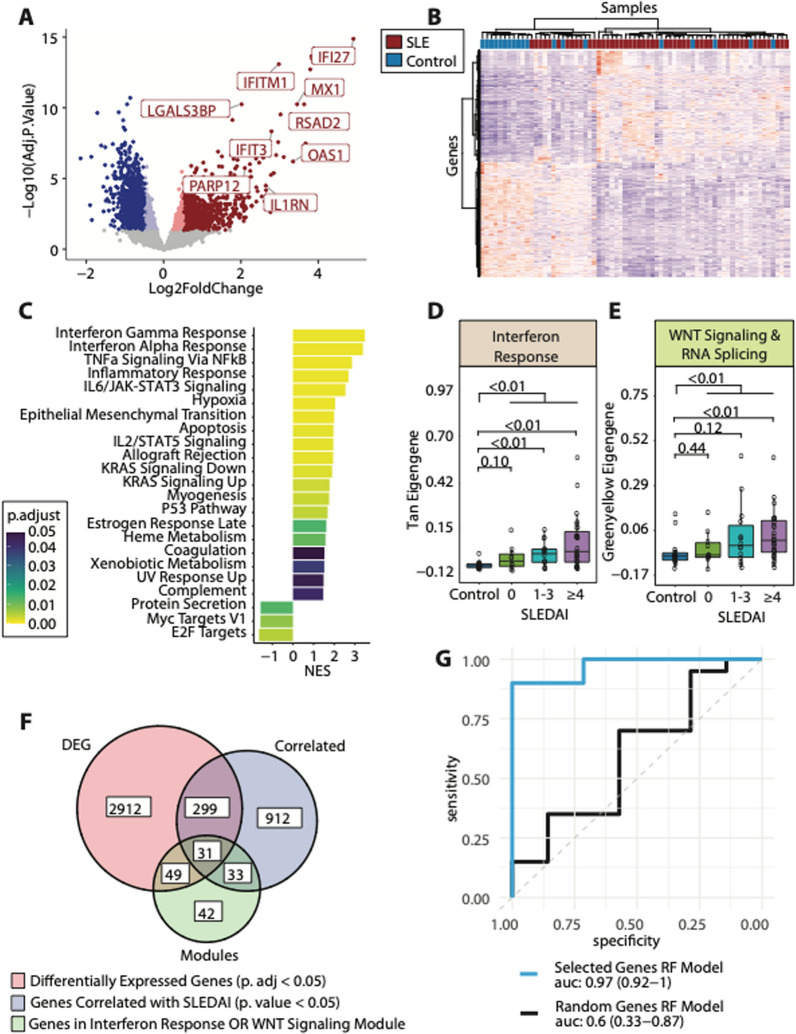
The Platelet Transcriptome is Significantly Different in SLE Subjects vs Controls. **A** Volcano plot of the differential expression between SLE subjects and controls. Red dots are upregulated genes with adjusted p. value < 0.05, dark red dots also pass a threshold of a log2foldchange > 0.5. Blue dots are downregulated genes with adjusted p. value < 0.05, dark blue dots also pass a threshold of a log2foldchange < -0.5. **B** Heatmap demonstrating unsupervised clustering of differentially expressed genes resulting in separation of our cohort by disease status. **C** Gene set enrichment analysis for the HALLMARK MSigDB pathways using the differentially expressed genes between our SLE and control cohort. The x-axis shows the normalized enrichment score (NES), and the color of the bar shows the adjusted p. value for the enrichment value. **D** Gene module scores for the *Tan* (interferon response) and the **E** *Greenyellow* (WNT Signaling and RNA splicing) modules across our entire cohort of patients. **F** Venn diagram of our feature selection process for genes to use in our machine learning model. We selected for genes that were differentially expressed (adjusted p. value < 0.05, red circle), genes with transcription correlated with SLEDAI values (p. value < 0.05, blue circle), and genes that belonged in either our *Tan* or *Greenyellow* modules (green circle). **G** ROC curves for a Random Forest model using our selected gene set [Blue line, AUC: 0.97 (0.92–1)] and a Random Forest model using a random equivalently sized gene set [Black line, AUC: 0.6, (0.33–0.87)]

Initial analyses focused on differences between the overall SLE cohort vs control samples. Evaluation of the platelet transcriptome between SLE and controls identified 2330 genes differentially expressed between groups (adjusted p value < 0.05, |log2FC|> 0.5, 1175 upregulated, 1155 downregulated, Fig. 2A, Additional file 3: Table S2). Many of the differentially expressed genes were upregulated interferon related (*IFI27, IFITM1, MX1, RSAD2, IFIT3, OAS1*) and other immunoregulatory genes (*LGALS3BP*, *IL1RN*, *PARP12*). Unsupervised clustering analysis using these differentially expressed genes was used to confirm the discernability of the lupus platelet transcriptome from the healthy one (Fig. 2B).

GSEA was performed on the differentially expressed genes. There were several enriched immune related pathways; interferon Gamma Response and Interferon Alpha Response were the most enriched pathways (NES = 3.48, adj. p = 0.001; NES = 2.86, adj. p value = 0.001 respectively). TNFa Signaling, IL6/JAK STAT3 Signaling, and Inflammatory Response were also all enriched in SLE versus controls. Notably, relevant to platelet functional responses, the pathway for Coagulation was enriched in SLE, but more modestly than those related to interferon response and immune activation (NES = 1.52, adjusted p value = 0.047; Fig. 2C).

When analyzing the modular framework, there were two modules that were (1) significantly different between SLE and controls, and (2) significantly correlated with disease activity as measured by SLEDAI. Both the interferon response (*Tan*) and the WNT Signaling and RNA splicing (*Greenyellow*) modules were significantly different between SLE and controls (p value = 2.67e-6) and significantly correlated with SLEDAI (*Tan:* R = 0.41, P = 0.003; *Greenyellow*: R = 0.33, P = 0.02; Fig. 1C, Fig. 2D, E).

To investigate the value of the platelet transcriptome in distinguishing participants with SLE from controls, we employed machine learning algorithms. The selected panel of genes were those that fulfilled the following criteria: (1) differentially expressed between SLE and controls (adjusted p value < 0.05, N = 3,291), (2) significantly correlated with SLEDAI (p < 0.05, |R|> 0.27, N = 1275), and (3) included in the interferon response (*Tan*) or the WNT Signaling and RNA splicing (*Greenyellow*) module (N = 155). Thirty-one genes met all these criteria and were selected for our confirmatory machine learning analysis (Fig. 2F; Additional file 4: Table S3). We used 60/40 balanced splits that were split into 10 random folds and followed by a fivefold cross validation. For this SLE-specific algorithm, accuracy was 0.89. and the area under the curve was 0.97 (95% confidence interval 0.92 to 1.00; Fig. 2G). Other models are included in Additional file 1: Fig S3A. In contrast, a model with thirty-one randomly selected genes performed significantly more poorly (AUC: 0.6 (0.33–0.87), Fig. 2F, Additional file 1: Fig S3B).

### Subjects with proteinuria have a distinct platelet transcriptome

**Fig. 3:**
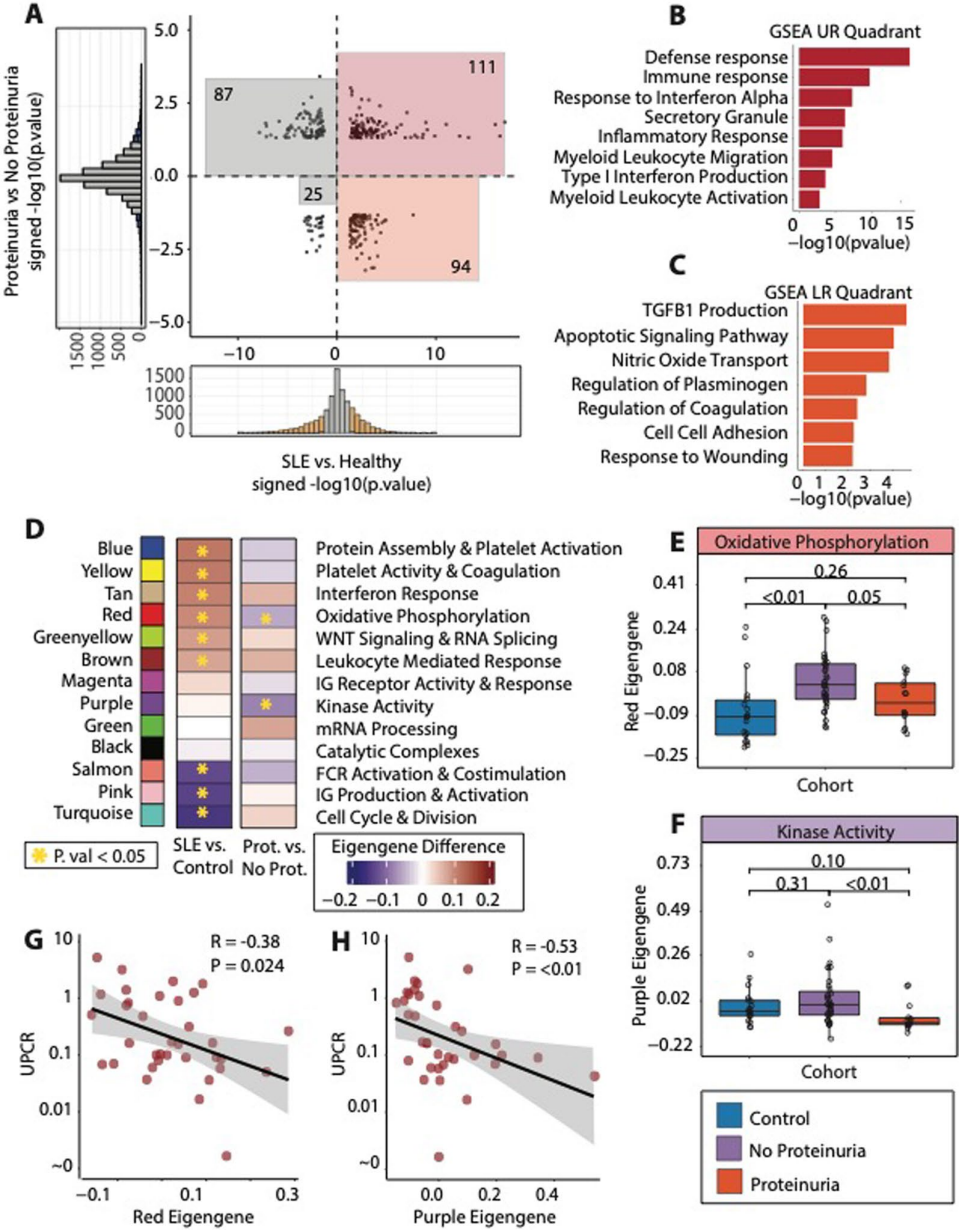
Lupus Subjects with Proteinuria have a distinct Transcriptome from a Generalized Lupus Signature **A** Scatterplot showing the signed -log10(p. value) values for the differential expression of SLE vs control (x-axis) and the differential expression of lupus patients with proteinuria vs lupus patients without proteinuria as defined by UPCR > 0.5. Genes in the red square are up in both SLE vs control, and proteinuria vs no proteinuria (N = 111), while genes in the orange square are up in SLE vs control, but down in proteinuria vs no proteinuria (N = 94). **B** Gene set enrichment analysis for genes that were up in SLE vs control and *up* in proteinuria vs no proteinuria. **C** Gene set enrichment analysis for genes that were up in SLE vs control and *down* in proteinuria vs no proteinuria. **D** Gene module framework with values demonstrating the difference in average module expression for SLE vs control, and proteinuria vs no proteinuria. Boxes annotated with a yellow star are those modules that were significantly different in their respective comparison. **E** Gene module scores for the *Red* (Oxidative Phosphorylation) and the **E** *Purple* (Kinase Activity) modules across our entire cohort of patients, annotated as controls, SLE patients with no proteinuria, or SLE patients with proteinuria. **G**, **H** are scatterplots comparing UPCR values vs the transcription of the *Red* and *Purple* gene modules

While we observed a clear signature that differentiated participants with SLE from controls, one of the major challenges in SLE is clinical heterogeneity. Proteinuria in the context of lupus nephritis is one of the most common and severe manifestations of SLE. We therefore focused our analysis on those with proteinuria at the time of blood draw as defined by a UPCR > 0.5 on random or 24 h collection. First, we performed differential expression analysis between SLE patients with versus without proteinuria. (Additional file 5: Table S4, Additional file 1: Fig S4A) We then compared this signature with the SLE overall signature derived previously in Fig. 2B. (Fig. 3A). Genes that were increased in SLE (vs control) and in those with proteinuria (compared to those without) are found in the upper right quadrant (N = 111, red). Genes that were increased in SLE but decreased in proteinuria are in the lower right quadrant (N = 94, orange). GSEA of the genes that were up in SLE regardless of proteinuria were enriched for pathways related to immune effector processes and interferon response and signaling (Fig. 3B). In contrast, genes that were up in SLE, but *down* in active proteinuria, were enriched for pathways related to regulation of coagulation and plasminogen, and TGFB1 (Transforming Growth Factor-Beta 1) production.

Consistent findings were seen in our modular framework. Figure 3D shows the differential values for each module when comparing SLE versus controls, and SLE with versus without proteinuria. As shown previously, when comparing transcriptomic modules between all our SLE participants and controls, there are many differentially regulated modules, with some key upregulated modules enriched for pathways involved in platelet activation, coagulation, and interferon response. When comparing SLE with versus without proteinuria, two modules indicative of platelet activation, oxidative phosphorylation (*Red*) and kinase activity (*Purple*) are both *decreased* in lupus patients with proteinuria. (Fig. 3E, F). Moreover, both the oxidative phosphorylation and kinase activity modular expression, are negatively correlated with the level of proteinuria (Fig. 3G, H).

### The low-binding FcγRIIA recessive allele associates with a decrease in FcγRIIA signaling pathways and clinical activity

Accumulating data demonstrates the contributing role of the FcγRIIA in platelet activity, impaired vascular health, and disease activity in patients with SLE [20, 21]. The low-binding H131R variant in subjects with SLE has been associated with worse disease outcomes, and consistently, 12 of the 13 participants with proteinuria in our cohort carried a low binding H131R allele. Of note, possession of the low binding H131R variant allele was observed in 39 of 51 (76%) participants with SLE and 14 of 18 (78%) controls (fisher test p value: 0.15).

**Fig. 4:**
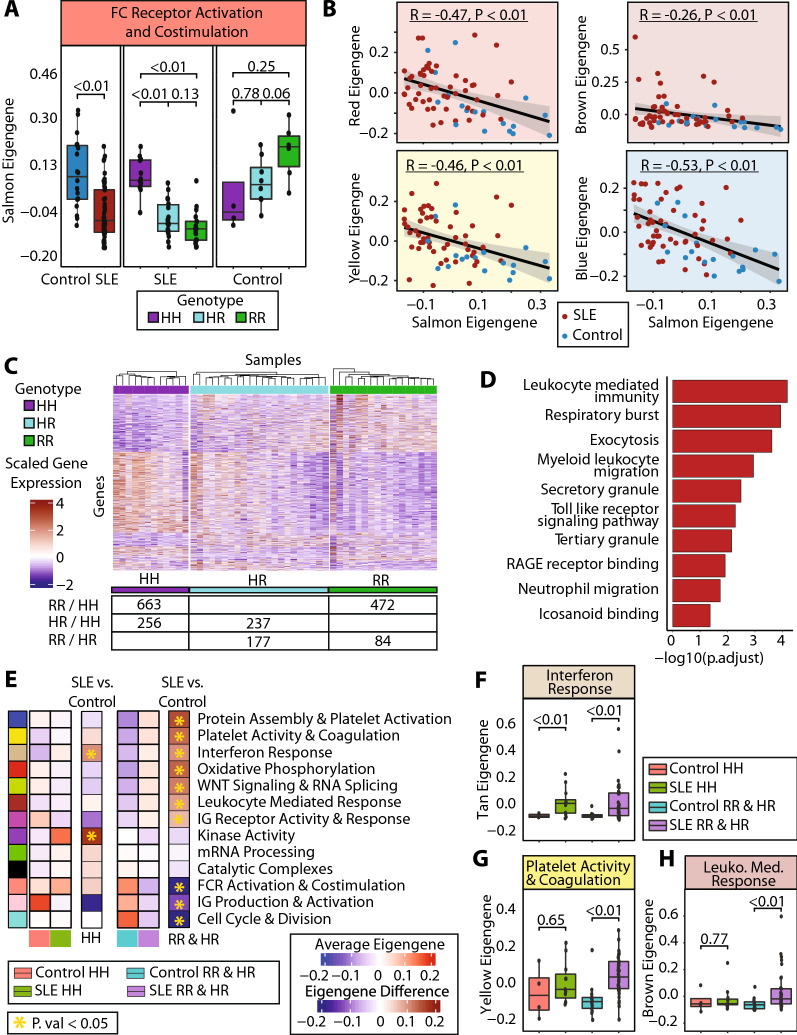
The Low-Binding FcγRIIA Recessive Allele Decreases FcγRIIA Signaling Pathways Leading to exacerbated SLE phenotypes **A** Gene module scores for the *Salmon* (FC Receptor Activation and Costimulation) for our cohort when divided by SLE vs control, and the SLE when split by genotype and Controls when split by genotype. **B** Correlation of our *Salmon* gene module expression vs the *Red* (Oxidative Phosphorylation), *Brown* (Leukocyte Mediated Response), *Yellow* (Platelet Activity and Coagulation), and *Blue* (Protein Assembly and Localization) gene modules. **C** Heatmap of SLE samples and genes differentially expressed when comparing HH vs RR, HH vs HR, and HR vs RR. The number of genes differentially expressed in each comparison are shown in the accompanying subtable (RR vs HH, 663 genes up in HH, 472 genes up in RR; HR vs HH, 256 genes up in HH, 237 genes up in HR; RR vs HR, 177 genes up in HR, 84 genes up in RR, p. value < 0.05 for all comparisons). **D** Hypergeometric gene set enrichment analysis for the genes that were increased in either RR vs HH or HR vs HH. **E** Gene module framework divided by disease status and FcγRIIA genotype. Comparison columns show the difference in average module expression for SLE vs control separated by the respective genotype, with significant differences annotated by a gold star. **F**–**H** show gene module scores for the *Tan*, *Yellow*, and the *Brown* gene module respectively across our cohort when divided by disease status and FcγRIIA allele status

The FC Receptor activation and costimulation module (*Salmon*) was significantly downregulated in SLE versus controls (p value = 2.88e-4). However, there was no association between this module and SLEDAI (Additional file 1: Fig S5A, B). When our population was stratified by FcγRIIA genotype, a different observation was noted for participants with SLE than controls. SLE subjects showed a significant decrease in the FC Receptor activation gene module when they possessed one or two copies of the low binding R variant allele (p value = 1.17e-6 for HR vs HH; p value = 9.46e-8 for RR vs HH).In contrast, there were no significant differences in the FCR activation gene module based on genotype among controls. (Fig. 4A). Exploration of the *salmon* eigengene demonstrates strong consistency of signal for each of the individual genes as well. With regards specifically to FCR activity, it is noted that *FYN* and *LYN*, which are key regulators in FCR signaling [43], are both significantly altered by the variant allele in SLE patients, but not significantly so in control patients (Additional file 1: Fig S5C).

The FC Receptor activation and costimulation module (*Salmon*) showed a negative correlation with other modules. The FC Receptor activation (*Salmon)* module was significantly inversely correlated with the oxidative phosphorylation module (*Red*), leukocyte mediated response module (*Brown*), platelet activity and coagulation module (*Yellow*) and protein assembly and localization module (*Blue*) (Fig. 4B).

We performed differential expression in our SLE cohort using pairwise comparisons of the FcγRIIA genotype: homozygous ancestral high-binding allele (HH), heterozygous variant low-binding allele (HR), and homozygous variant low-binding allele (RR). Consistent with variant dominance biology, the biggest differences in the platelet transcriptome were observed when comparing those with the RR allele vs those with the HH allele (1,135 differentially expressed genes, p value < 0.05), followed by HR vs HH (493 differentially expressed genes) and finally RR vs HR (261 differentially expressed genes) (Fig. 4C). When we performed GSEA on genes that were increased in participants with at least one copy of the low binding R variant allele (either RR vs HH, or HR vs HH), we found strong enrichment for immune related pathways, such as leukocyte mediated response, respiratory burst, and RAGE Receptor Binding (adjusted p value < 0.05) (Fig. 4D).

Our modular framework stratified by SLE versus control, and by FcγRIIA genotype is noted in Fig. 4E. Among SLE and controls homozygous for the high-binding ancestral allele (HH), many of the differences previously observed in SLE versus controls are no longer present. The only significantly difference between SLE and controls is the interferon signaling (*Tan*) module (p value =  < 0.01, Fig. 4F). In contrast, among SLE and controls with at least one copy of the low-binding variant allele, many differences emerge. This is exemplified by the platelet activity and coagulation (*Yellow*) and leukocyte mediated response (*Brown*) modules. While both modules are not different between SLE and controls carrying HH for FcγRIIA (p = 0.65 for *Yellow*, p = 0.77 for *Brown*), there are significant differences in these modules between SLE and controls carrying at least one copy of the low binding allele (p = 2.03e-4 for *Yellow*, p = 1.38e-3 for *Brown*) (Fig. 4G). An exploration of interaction between SLE diagnosis and the possession of a variant allele was performed using a ranked linear model that included an interaction term for SLE and an FCGR2a variant. While limited by population size to perform such an analysis, we see similar results with alterations in platelet activation pathways occurring most significantly in patients with both SLE and a variant allele (Additional file 1: Fig S5D).

These data reinforce an important synergistic effect of the FcγRIIA variant. While SLE patients have upregulation of interferon activity regardless of FcγRIIA status, among individuals with the FcγRIIA low binding allele, a robust transcriptomic shift with upregulation in many inflammatory pathways, including platelet activity and immune activation, was observed.

### A novel platelet transcriptomic score associated with a change in disease activity

**Fig. 5:**
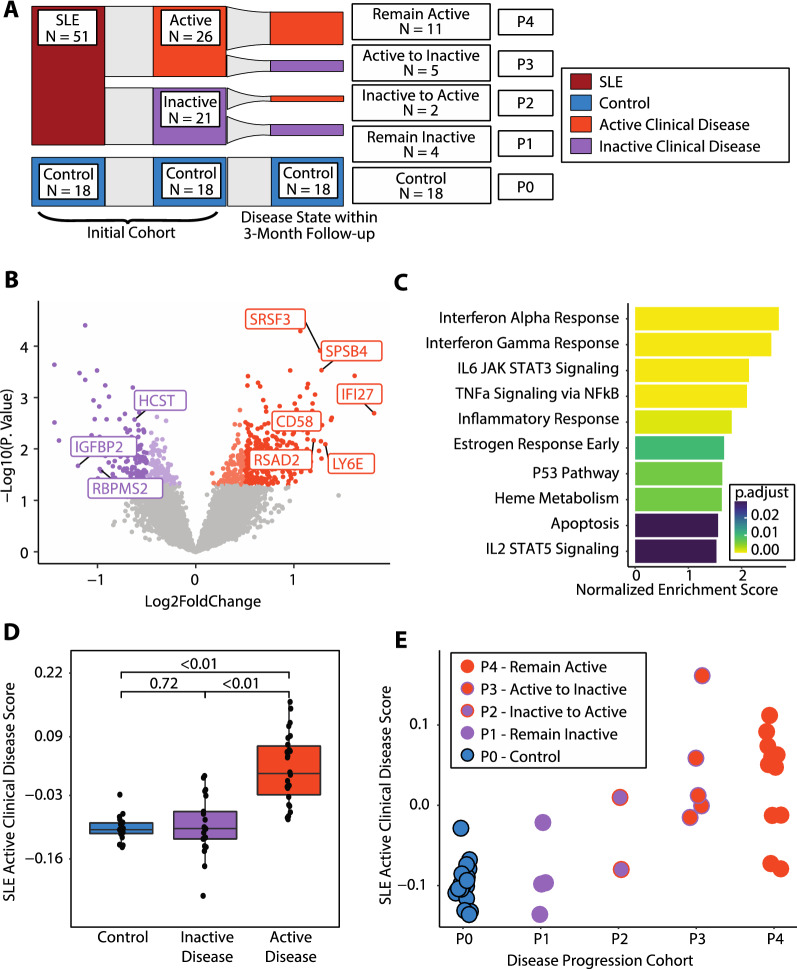
A Novel Platelet Transcriptomic Score associated with a change in Lupus Activity **A** Sankey diagram showing our cohort at the initial timepoint by disease status, active clinical disease status, and then active clinical disease status within 3 months of follow-up. Controls are P0, P1 are patients that remained inactive (N = 4), P2 are patients that had clinical flares of disease (N = 2), P3 are patients that improved disease activity (N = 4), and P4 are patients that remained active (N = 11). **B** Volcano plot for the differential expression between SLE patients with active clinical disease vs no active clinical disease. **C** GSEA for the differential expression of active clinical disease vs no active clinical disease. **D** SLE Active Clinical Disease Score for our cohort, divided into controls, and patients with inactive clinical disease and active clinical disease. **E** Scatterplot of patients showing a correlation between the disease progression cohort and the SLE Active Clinical Disease Score. Blue dots are control patients (P0), purple dots are patients with inactive disease at point of collection (P1), purple dots with orange outlines are patients who were inactive at collection, but transitioned to active clinical disease within 3 months (P2), orange dots with purple outlines are patients who with active clinical disease at baseline and transitioned to inactive clinical disease within 3 months (P3), and solid orange dots are patients with active clinical disease at the point of sample collection and remained active within the 3 month follow-up (P4)

Tracking clinical manifestations of SLE activity and pairing clinically relevant disease and shifts in disease state to transcriptomic data may aid in the understanding and prediction of SLE progression. To assess whether the platelet transcriptome predicts SLE clinical activity changes in an intermediate timeframe, patients were categorized based on their dominant SLEDAI domain at the time of the blood draw and follow-up within 3 months if clinically available. (Fig. 5A).

Using the predominant disease categories defined within the Methods section, we classified SLE patients into two clinical disease categories: (1) *inactive* clinical disease (defined by no clinical SLEDAI domain but could be serologically active with anti-DNA and/or low complements); and (2) *active* clinical disease (defined by clinical symptoms such as rash, arthritis, nephritis, or any other clinical SLEDAI domain). We performed differential expression between SLE participants with *active* clinical disease (N = 26) versus *inactive* clinical disease (N = 21) at the time of blood draw. There were significant differences in the platelet transcriptome between active versus inactive SLE (397 upregulated genes, 279 downregulated genes, p value < 0.05, Fig. 5B). Differential analysis results were not significantly altered when drugs were included as covariates in the model. GSEA on these differentially expressed genes showed pathways consistent with the defined SLE signature, with enriched pathways of interferon response, TNFa Signaling via NFkB, and inflammatory response in participants with *active* clinical disease (Fig. 5C).

Next, we created a single value representative of an SLE *active* clinical disease signature using singscore based on the differentially expressed genes, where we used our 397 upregulated genes as the “upSet” and the 279 downregulated genes as the “downSet” [40] (Additional file 6: Table S5). To test the applicability of the collapsed score, we reapplied the singscore-developed active clinical disease score to our cohort. The score was significantly different between patients with *active* clinical disease vs inactive clinical disease (p value < 2.30e-7; Fig. 5D). Notably, when the signature was applied to our control patients (not involved in the derivation of the signature), there was no significant difference between patients without active clinical disease at the time of blood draw and healthy controls. (p = 0.72; Fig. 5D).

Twenty-one SLE participants had their clinical disease assessed at baseline and within 3 months of follow-up. Among 15 SLE participants with *active* clinical disease at baseline, 11 remained active and 5 transitioned to inactive (the clinical domain was no longer present). Of the 6 SLE participants who were clinically inactive at baseline, 4 remained inactive and 2 experienced a clinical flare and transitioned to active (Fig. 5A). We are limited by the number of patients that fit into these distinct disease progression cohorts to perform pairwise comparisons; however, we note that the SLE *active* clinical disease score was correlated with changes in disease activity. (Fig. 5E).

## Discussion

The application of sequencing technology to blood and its components is a means of applying cutting-edge technology to help answer fundamental questions. Pathology in SLE is complex and heterogenous, and the flaring of disease coupled with its complex presentation makes understanding its pathophysiology a significant challenge. Platelets confer important contributions to inflammation and immune regulation, and their well described role in vascular homeostasis, means that these circulating cells may inform our understanding of SLE.

### Beyond interferon signaling in SLE pathology

It has been known for over a decade that patients with SLE exhibit evidence of type I Interferon signaling as reflected by increased Interferon Stimulated Genes (ISGs) in peripheral blood monocytes and elevated levels of the cytokine being produced by cells and measured across various tissues [44–46]. These results are mirrored in our platelet transcriptomics, where SLE subjects showed upregulation of interferon related genes (*IFITM1, IFI27, RSAD2, IFIT3, LGALS3BP*) and the most enriched for pathways amongst the differentially expressed genes were interferon gamma response and interferon alpha response. Furthermore, when our platelet transcriptome was labeled via our novel WGCNA framework, one of the most consistently differentially regulated gene modules was our *Tan* module, the module most enriched for interferon response.

Our transcriptomic analysis also demonstrated significant dysregulation in other immune related pathways such as *TNFa Signaling *via NFkB, *Inflammatory Response*, and *IL6/JAK-STAT3 Signaling*. The modular framework, described above, allows us to analyze interferon signaling with a specific module (*Tan*), while also being able to explore other effects that SLE has on the platelet transcriptome via other pathways and modules. So, while IFN activity and response are a strong and consistent signal in SLE, it is clear from our analyses that we can parse and analyze several other immunoregulatory pathways, and platelet specific activation and vascular homeostatic pathways.

### The changing behaviors of platelet transcriptomics with regard to lupus patients with proteinuria

The shifting results regarding enrichment of pathways involved in coagulation are of particular interest. We see that in the initial exploration of SLE vs controls, the coagulation pathway is positively enriched in SLE. However, it is notable that its enrichment is less significant when compared to other immune pathways. This suggests a possible heterogeneous effect; while immune dysregulation is present in all SLE patients, there are subpopulations that exhibit different changes in the transcriptional profiles related to coagulation, which leads to the lesser enrichment of this pathway. This heterogeneity of disease was supported when the transcriptome of our active lupus was explored in patients with proteinuria. Our analysis demonstrates that genes that are highly expressed in SLE vs controls and increased in SLE patients with versus without proteinuria, are enriched for the kind of classical SLE pathology seen previously: interferon response signaling, lymphocyte migratory signaling, and NFkB signaling. But the genes enriched in SLE, yet *down* in lupus patients with proteinuria included those in coagulation and wound response related pathways.

Taken together with the fact that the gene modules enriched for oxidative phosphorylation and kinase activity, pathways often associated with activation, are downregulated in our lupus patients with proteinuria, an emerging picture supports that that in patients with proteinuria there is a *decrease* in many platelet transcriptomic activity pathways. While pathways related to coagulation and platelet activity are increased in our SLE population, there is a surprising reversal in platelet activity modules in patients with proteinuria, such that when patients evolve to this more severe manifestation, there is a *decrease* in platelet activation pathways. It remains unknown if this is a compensatory change or a premonitory change. Due to the complexity of medication dosage and variety, it is difficult to separate out drug effects. Notably though, medication use was not statistically significantly different between those lupus patients with proteinuria and those without. Nevertheless, we performed differential analysis that included medication use as covariates, and the results showed a very similar signature of differential expression (correlation R value of log2FC values = 0.97, data not shown). Furthermore, this shift has been reported clinically as well, where the type of chronic platelet activation that is seen in patients with SLE leading to platelet exhaustion [47].

The validation of omic data with phenotypic response creates a complete picture that may help explain SLE pathophysiology from molecular underpinnings to actionable biomechanisms. Demonstrating that the SLE modules decreased in lupus patients with proteinuria also correlated with the level of proteinuria levels suggests the potential for molecular profiling to better predict and track the activity of disease outside of traditional lab measures. Furthermore, the fact that modular transcription correlated with UPCR levels *below* the 0.5 level as well means that these modules may be able to track disease progression earlier and at subclinical levels, allowing for more immediate clinical action.

### The implications of FcγRIIA genotype in the disease activity and progression of SLE

FcγRIIA, encoded by the FCGR2a gene, is a key receptor for communication between platelets and the surrounding immunological milieu [17]. FcγRIIA binds the FC portion of antibodies, which then initiates a signaling pathway that creates a responsive role in platelets to their microenvironment. FcγRIIA allelic status has been established as a risk factor in the disease activity of lupus [48, 49]. Previous data from our group found an association between the low-binding variant alleles of FcγRIIA with platelet activity and subclinical CVD [21]. Our current analysis shows that the low-binding allele is associated with the expected effect of lower transcription of our module enriched for FCR activation (*salmon*). Interestingly, this model is inversely correlated with modules of platelet activation and immune response. Consistently, studies have observed that the variant low-binding allele is associated with poor clinical outcomes, activated platelets, and increased immune stimulation [21]. It follows that the low-binding allele would lead to less FC signaling cascade activity. In total, this illustrates a process of the low-binding allele leading to less FC signaling, which is then associated with increased pathway expression of platelet activation and immune response, which leads to worse disease.

Differential expression within our SLE cohort of patients who are homozygous ancestral (HH), heterozygous (HR) and homozygous variant (RR) illustrates a variant dominance effect, where RR patients were most different from HH subjects. Gene set enrichment of genes that were differentially expressed in subjects with at least one low-binding allele (HR or HH) versus subjects homozygous for the high-binding allele (RR) revealed enrichment for immune activation pathways, further showing that the low-binding variant associates with an increase in transcription of pathways related to immune activation.

Based on our findings, we stratified our modular transcriptomic framework by FCGR2a allelic status. The interferon module was increased in SLE regardless of FCGR2a status. However, differences in immune activation, platelet activation, and coagulation were only increased in SLE patients who possessed a low binding variant allele (HR or RR).

While this warrants further investigation, we posit two possible theories. The first one, which is less likely, is that the FC signaling cascade in platelets is in fact *inhibitory*, such that when a low-binding allele leads to less FC signaling, the inhibitory effect is lost which leads to activation of platelets and an increase immune response. Alternatively, a more likely possibility is that the low-binding allele is leading to less clearance of immune complexes. This increase in immune complex prevalence leads to activation of complement and platelets in their immune and vascular homeostatic roles. These changes are then reflected in in the transcriptome, and in the proceeding SLE sequelae in the form of worsening disease, and an increased inflammatory and immune activated state.

### Predicting the progression of SLE disease activity

It is well appreciated that the prediction of SLE flares would allow for more targeted management of disease, potentially leading to better outcomes in SLE. SLEDAI, despite being a validated instrument to assess SLE disease activity, is a point system in which domains are scored whether the clinical manifestation is severe or mild (e.g., near cardiac tamponade from pericarditis scores and simple alopecia both score as 2). To better appreciate clinical associations, we assessed patients according to different manifestations which dominated the overall clinical phenotype. This rating system allows clinicians to better assess the patient-centered chief complaint, as opposed to using a scoring system that does not consider the specific clinical implications of an individual’s presentation.

When we used this delineating system, we were able to create a signature of disease flare that was reduced to a single score. Interestingly, even though controls were not considered when deriving the score, this score was not significantly different between our healthy controls and SLE with inactive clinical disease. This would indicate that from a transcriptomic point of view, the signature captures genes that only change in the presence of clinical disease manifestations and are not different between SLE patients with no clinical disease, and healthy non-SLE controls. For the purposes of tracking active clinical disease, this was an intriguing preliminary finding suggesting a novel means of using the platelet transcriptome to capture SLE patients with clinical activity. Furthermore, this score showed a very significant difference in those that had active clinical disease vs those with inactive clinical disease.

We acknowledge that the finding of tracking flares of disease is underpowered with too few patients to make definitive statements. Regardless, the longitudinal follow-up available in our SLE cohort allowed for reassessment of disease state within 3 months and was available for 21 patients. The majority had no changes in their clinical disease status, but a subset of patients had changes in a clinical domain being scored or not scored at baseline and subsequent visit. When we assigned the various disease states on an ordinal scale, there was a robust correlation, with patients who changed their disease state falling in between those who remained active and those who remained inactive.

Altogether, these data support the potential for a platelet RNA-seq based scoring metric, which may distinguish patients not only who have active clinical SLE phenotypes vs those without but can potentially aid in determining whether SLE participants will flare or not based on the changes of their SLE Active Clinical disease score. Future studies will need to validate these findings and investigate whether clinical decision making should be modified based on this score.

### Limitations of the study

There are several limitations to acknowledge. Consistent with typical SLE presentation, [22] our cohort is exclusively female, future studies are required to assess if our findings are impacted by biologic sex. In addition, drug effects are always of concern in studies of subjects with autoimmune disease, and while medication use did not appear to confound our analyses, we were not powered to adequately incorporate medication use into each analysis. As far as the clinical manifestation of SLE, analyses were focused on the overall SLE signature, and lupus patients with proteinuria. The current cohort does not have sufficient patients with other manifestations such as arthritis since non-steroidal medications were an exclusion criterion for individualized analyses, but further enrollment will allow for a similar symptom specific analysis to be performed. Our final analysis where we analyzed differences between active and inactive clinical disease relied on nominal P values as the differences were more subtle than those seen when comparing SLE vs control. Lastly, we acknowledge the analysis with regards to patients changing clinical disease statement is underpowered.

## Conclusion

The complex pathophysiology of SLE makes elucidating the biomolecular underpinnings a challenge. Therefore, using cutting-edge technology such as RNA-seq on accessible blood derivatives such as platelets is an attractive avenue to gain insight into this disease. Platelets are readily accessible and play key roles in vascular homeostasis and endothelial health, both of which contribute to SLE pathophysiology.

These data support that platelet transcriptomics in SLE are not only significantly and widely different from healthy platelets, but also vary in nuanced ways with regards to specific disease manifestations such as proteinuria. Lupus patients with proteinuria, appear to demonstrate a paradoxical shift of their platelet transcriptomics with decreases in modules associated with platelet activation. Low-binding variants of FcγRIIA synergize with SLE to create more substantial transcriptomic changes, with implications of a low-binding FcγRIIA variant leading to less FC activation and overall being associated with increases in transcriptomic activity indicative of immune and platelet activation. Finally, a novel platelet transcriptome classification system can capture the disease state of each patient and can help in the effort to predict changes in disease activity.

## Supplementary Information

**Additional file 1: Figure S1. **Flow cytometry of platelet samples demonstrate levels of CD45 and Glycophorin A before (A) and after (B) depletion procedures. Transcript levels of platelet, white blood cell, and red blood cell enriched genes (C) as determined by RNA-seq.** Figure S2.** (A) Histogram depicting the filter of 8 reads in at least half of samples used to select for genes that were considered significantly expressed. (B) Dotplot showing a selected subset of the gene sets used for annotation of the gene modules. (C) Heatmap where the expression of each module is shown for each sample. Samples are hierarchically clustered and annotated by cohort. **Figure S3.** (A) ROC curves for the 5 models tried (GLM, xgBoost, SVM, Random Forest, and Lasso) using our selected gene set. (B) ROC curves for the 5 models tried (GLM, xgBoost, SVM, Random Forest, and Lasso) using a randomly selected set of genes of equivalent size to our selected gene set. **Figure S4.** (A) Volcano plot showing the differential expression results of SLE patients with proteinuria vs SLE patients without proteinuria. **Figure S5.** (A) Gene module scores for the Salmon (FC Receptor Activation and Costimulation) for our cohort when divided by SLEDAI range for the respective SLE patients. (B) Salmon eigengene values vs SLEDAI values for the SLE cohort. Correlations were calculated for the entire SLE cohort (black), SLE patients who are homozygous ancestral (HH, green), and patients who were heterozygous (HR) and homozygous variant (RR) (purple). (C) Heatmaps of the individual genes that make up the Salmon gene module separated into the SLE and control cohort, and annotated by the FcγRIIA genotype. Annotation columns are included to show whether or not the individual genes are differentially expressed for each respective comparison. (D) Forest plot for the coefficient value of each term when modeling diagnosis and variant allele against eigengene expression. Asterisked colors indicated significant association of the feature with the eigengene.

**Additional file 2: Table S1.** Table with the results of GO term enrichment for each of the gene modules output from WGCNA. Columns include the module/GO term combination, then the module, the number of genes in the module, the p .value and adjusted p. value for the enrichment test of the GO term within the module, the gene ratio for the enrichment test, the ontology database used, and then the ID of the GO term.

**Additional file 3: Table S2.** Table with the results of the differential expression analysis comparing the SLE cohort with the control cohort. The columns are the gene; the average expression of the gene across all samples; the log2foldchange between the two groups with positive values indicating increase in the SLE cohort; the standard error of the log2foldchange; the Wald statistic; and then the p. value and the adjusted p. value for the comparison.

**Additional file 4: Table S3.** The genes selected for machine learning for the prediction between SLE and control that filled the criteria of (1) Differentially expressed between SLE and control, (2) correlated with the SLEDAI values of patients, and (3) part of the *greenyellow* or *tan* module.

**Additional file 5: Table S4.** Table with the results of the differential expression analysis comparing the patients with proteinuria vs those without within the SLE cohort. The columns are the gene; the average expression of the gene across all samples; the log2foldchange between the two groups with positive values indicating increase in the proteinuria cohort; the standard error of the log2foldchange; the Wald statistic; and then the p. value and the adjusted p. value for the comparison.

**Additional file 6: Table S5.** Table with two tabs, one for the “Upset” and one for the “Downset” that make up the genes that go into the singscore algorithm to derive the SLE active clinical disease score.

## Data Availability

Sequencing data is available on GEO GSE226147. All other output is supplied as additional file information. Additional data, results, or code available upon request.
