# Supplementary material for: Modeling of clinical phenotypes in systemic lupus erythematosus based on the platelet transcriptome and FCGR2a genotype
Source: J Transl Med. 2023 Apr 7;21:247. doi: 10.1186/s12967-023-04059-w (PMC10082503; doi:10.1186/s12967-023-04059-w)
Supplement: Supplementary file 1 — Additional file 1: Figure S1. Flow cytometry of platelet samples demonstrate levels of CD45 and Glycophorin A before (A) and after (B) depletion procedures. Transcript levels of platelet, white blood cell, and red blood cell enriched genes (C) as determined by RNA-seq. Figure S2. (A) Histogram depicting the filter of 8 reads in at least half of samples used to select for genes that were considered significantly expressed. (B) Dotplot showing a selected subset of the gene sets used for annotation of the gene modules. (C) Heatmap where the expression of each module is shown for each sample. Samples are hierarchically clustered and annotated by cohort. Figure S3. (A) ROC curves for the 5 models tried (GLM, xgBoost, SVM, Random Forest, and Lasso) using our selected gene set. (B) ROC curves for the 5 models tried (GLM, xgBoost, SVM, Random Forest, and Lasso) using a randomly selected set of genes of equivalent size to our selected gene set. Figure S4. (A) Volcano plot showing the differential expression results of SLE patients with proteinuria vs SLE patients without proteinuria. Figure S5. (A) Gene module scores for the Salmon (FC Receptor Activation and Costimulation) for our cohort when divided by SLEDAI range for the respective SLE patients. (B) Salmon eigengene values vs SLEDAI values for the SLE cohort. Correlations were calculated for the entire SLE cohort (black), SLE patients who are homozygous ancestral (HH, green), and patients who were heterozygous (HR) and homozygous variant (RR) (purple). (C) Heatmaps of the individual genes that make up the Salmon gene module separated into the SLE and control cohort, and annotated by the FcγRIIA genotype. Annotation columns are included to show whether or not the individual genes are differentially expressed for each respective comparison. (D) Forest plot for the coefficient value of each term when modeling diagnosis and variant allele against eigengene expression. Asterisked colors indicated significant association of th [file 12967_2023_4059_MOESM1_ESM.docx]

Additional Materials for

**Modeling of Clinical Phenotypes in Systemic Lupus Erythematosus Based on the Platelet Transcriptome and FCGR2a Genotype**

MacIntosh G. Cornwell^1,2^, Hanane El Bannoudi^3^, Elliot Luttrell-Williams^3^, Alexis Engel^4^, Tessa J. Barrett^3,5^, Khrystyna Myndzar^4^, Peter Izmirly^4^, H. Michael Belmont^4^, Robert Clancy^4^, Kelly V. Ruggles^1,2^*, Jill P. Buyon^4^*, Jeffrey S. Berger^3,5^*

*Corresponding author.

**Corresponding Authors**:

Jeffrey S. Berger, MD, MS

E-mail: jeffrey.berger@nyulangone.org

Jill P. Buyon, MD

Email: [jill.buyon@nyulangone.org](mailto:jill.buyon@nyulangone.org)

Kelly V. Ruggles

Email: Kelly.ruggles@nyulangone.org

**This PDF file includes:**

Figs. S1 to S5

Tables S1 to S5

Supplementary Figures


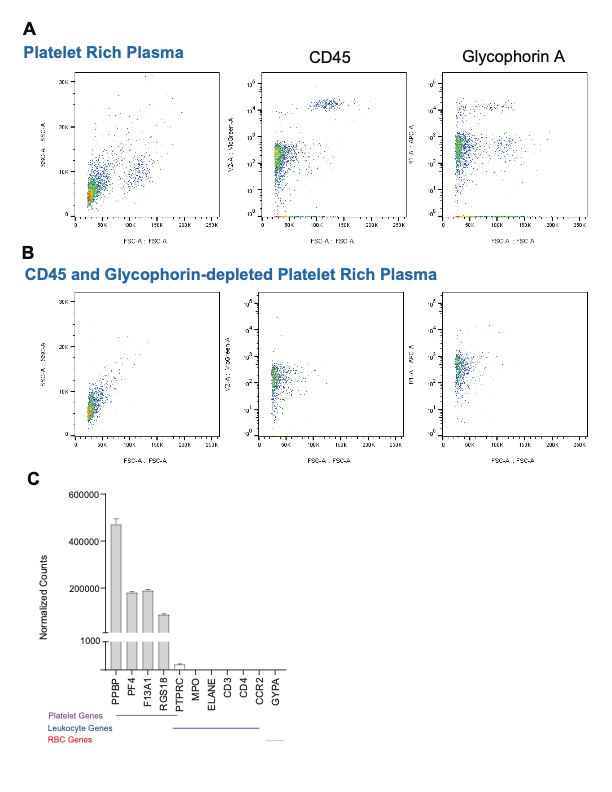


Supplemental Figure 1: Flow cytometry of platelet samples demonstrate levels of CD45 and Glycophorin A before (A) and after (B) depletion procedures. Transcript levels of platelet, white blood cell, and red blood cell enriched genes (C) as determined by RNA-seq.

**
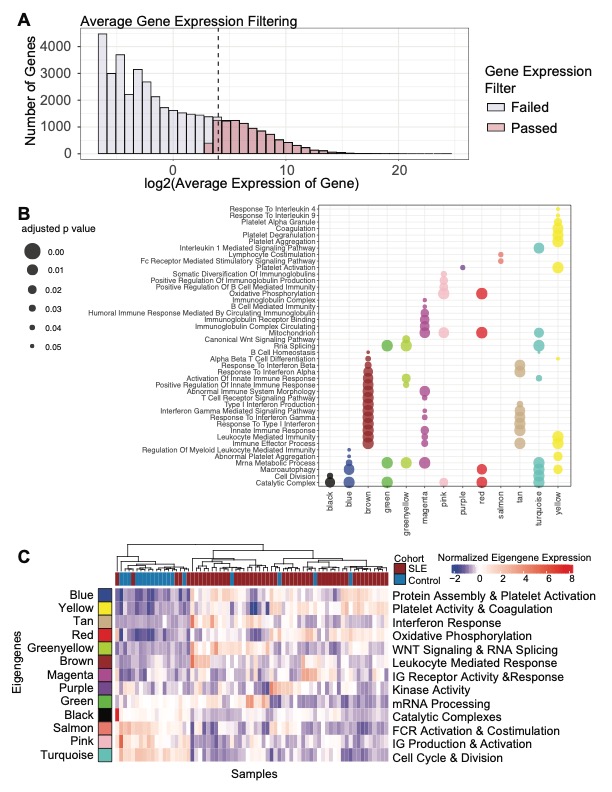
**

**Supplemental Figure 2:** (A) Histogram depicting the filter of 8 reads in at least half of samples used to select for genes that were considered significantly expressed. (B) Dotplot showing a selected subset of the gene sets used for annotation of the gene modules. (C) Heatmap where the expression of each module is shown for each sample. Samples are hierarchically clustered and annotated by cohort.

**
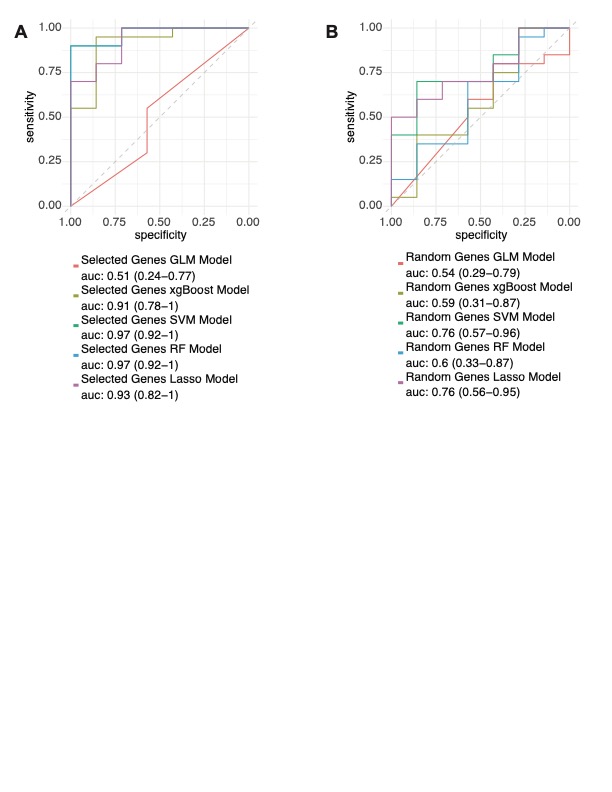
**

**Supplemental Figure 3:** (A) ROC curves for the 5 models tried (GLM, xgBoost, SVM, Random Forest, and Lasso) using our selected gene set. (B) ROC curves for the 5 models tried (GLM, xgBoost, SVM, Random Forest, and Lasso) using a randomly selected set of genes of equivalent size to our selected gene set.

**Supplemental Figure 4:** (A) Volcano plot showing the differential expression results of SLE patients with proteinuria vs SLE patients without proteinuria.


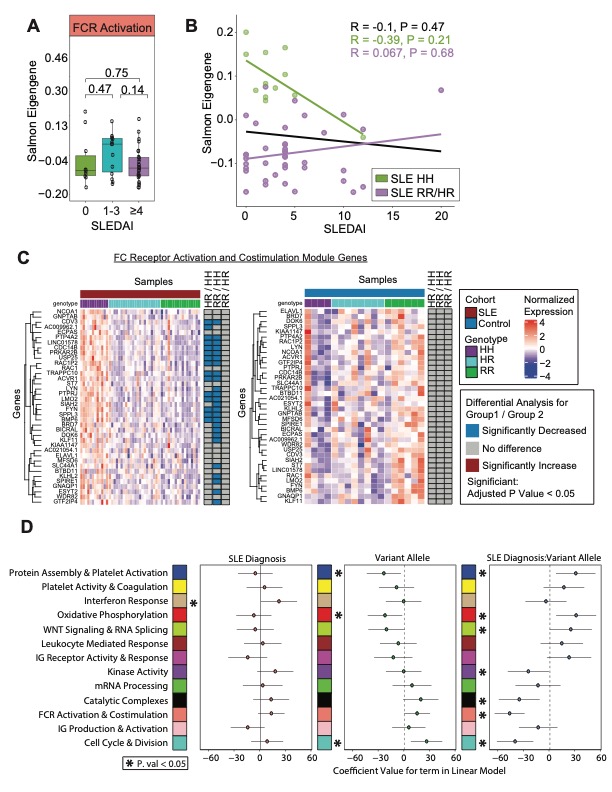


**Supplemental Figure 5:** (A) Gene module scores for the *Salmon* (FC Receptor Activation and Costimulation) for our cohort when divided by SLEDAI range for the respective SLE patients. (B) *Salmon* eigengene values vs SLEDAI values for the SLE cohort. Correlations were calculated for the entire SLE cohort (black), SLE patients who are homozygous ancestral (HH, green), and patients who were heterozygous (HR) and homozygous variant (RR) (purple). (C) Heatmaps of the individual genes that make up the *Salmon* gene module separated into the SLE and control cohort, and annotated by the FcγRIIA genotype. Annotation columns are included to show whether or not the individual genes are differentially expressed for each respective comparison. (D) Forest plot for the coefficient value of each term when modeling diagnosis and variant allele against eigengene expression. Asterisked colors indicated significant association of the feature with the eigengene.

**Supplemental Tables:**

**Supplemental Table 1:** (Supp_Tab_1_C5_wgcna_module_annotations.csv) Table with the results of GO term enrichment for each of the gene modules output from WGCNA. Columns include the module/GO term combination, then the module, the number of genes in the module, the p .value and adjusted p. value for the enrichment test of the GO term within the module, the gene ratio for the enrichment test, the ontology database used, and then the ID of the GO term.

**Supplemental Table 2:** (Supp_Tab_2_deseq_results_comp_sle__sle_v_control) Table with the results of the differential expression analysis comparing the SLE cohort with the control cohort. The columns are the gene; the average expression of the gene across all samples; the log2foldchange between the two groups with positive values indicating increase in the SLE cohort; the standard error of the log2foldchange; the Wald statistic; and then the p. value and the adjusted p. value for the comparison.

**Supplemental Table 3:** The genes selected for machine learning for the prediction between SLE and control that filled the criteria of (1) Differentially expressed between SLE and control, (2) correlated with the SLEDAI values of patients, and (3) part of the *greenyellow* or *tan* module.

**Supplemental Table 4:** (Supp_Tab_4_deseq_results_comp_proteinuria__prot_v_noprot.csv) Table with the results of the differential expression analysis comparing the patients with proteinuria vs those without within the SLE cohort. The columns are the gene; the average expression of the gene across all samples; the log2foldchange between the two groups with positive values indicating increase in the proteinuria cohort; the standard error of the log2foldchange; the Wald statistic; and then the p. value and the adjusted p. value for the comparison.

**Supplemental Table 5:** (Supp_Tab_5_SLE_active_clinical_disease_genesets.xlsx) Table with two tabs, one for the “Upset” and one for the “Downset” that make up the genes that go into the singscore algorithm to derive the SLE active clinical disease score.
